# Positive Emotions Training (PoET) as an online intervention to improve mental health: a feasibility study

**DOI:** 10.1186/s12889-023-16424-x

**Published:** 2023-08-14

**Authors:** Lara Niemann, Celin von Gruner, Xiao Chi Zhang, Jürgen Margraf, Christina Totzeck

**Affiliations:** 1 Mental Health Research and Treatment Centre, Ruhr University Bochum, Massenbergstrasse 11, 44797 Bochum, Germany; 2 DZPG (German Center for Mental Health), Bochum/Marburg, Germany

**Keywords:** Online training, Positive emotions, Positive psychology, Mental health, Low-threshold intervention, Feasibility

## Abstract

**Background:**

Positive psychology interventions are known to have an impact on mental health as well as on a number of beneficial characteristics like optimism, gratitude and self-efficacy. The Positive Emotions Training (PoET) is one of the first holistic training programs covering eleven positive psychology constructs. The goal of this study was to test PoET’s feasibility in the general population and to assess possible effects on positive and negative mental health factors. Additionally, possible effects on optimism, gratitude, happiness, resilience, and self-efficacy were examined.

**Methods:**

The sample (*n* = 101) was not randomized. Participants were allocated to PoET (*n* = 55) or control group (*n* = 46) that did not receive treatment initially. The PoET group completed two training sessions (3.5 h each) that were conducted in an online format with groups of about 30 people. All participants completed positive and negative mental health measures at the beginning of the first training session and at the beginning of the second one as well as 30 days after the second session. Two-factorial repeated measures ANOVAs were conducted to test for possible effects of PoET on mental health.

**Results:**

The results showed that the contents were comprehensible and that the conduction of the training was feasible overall. In addition, a significant decrease of depression and anxiety symptoms as well as a significant increase of optimism were found in the PoET group. No significant changes were found in the control group.

**Conclusions:**

Results indicate that PoET is an applicable intervention for improving mental health in the general population.

**Trial registration:**

Retrospectively registered at ClinicalTrials.gov on 21/02/2023 (Identifier/Trial registration number: NCT05737251).

## Background

 Mental disorders affect millions of people worldwide, causing suffering and various negative consequences. According to the World Health Organization (WHO), the lifetime prevalence ranges from 18.1 to 36.1% [1]. Studies indicate reduced life expectancy by nearly 18 years for individuals with mental disorders [2–4]. Also, Disability Adjusted Life Years (DALYs) [5] related to mental disorders have significantly increased since 2005, with depressive and anxiety disorders ranking among the top causes [6]. Global public healthcare costs associated with mental disorders are substantial [7, 8]. For example, in the US anxiety disorders alone account for one-third of total expenditures on mental disorders [9, 10]. Depressive disorders in the UK result in £9 billion in costs, 100 million lost working days, and 2,615 deaths [11], while, for example, Germany spends approximately €27 billion on mental disorder treatment and compensation, representing 11.3% of medical spending [12]. In addition, mental disorders negatively impact education, employment, and overall quality of life [13]. These findings indicate that mental disorders have severe negative consequences for individuals and society.

Several studies highlight the psychological burden of the COVID-19 pandemic and related restrictions [14–16], including a negative impact on mental health in the general population [17–19]. Unlike other mental disorders that returned to pre-pandemic levels, depression and mood disorders remained alarmingly high [20]. Germany, specifically, faces overcrowded psychotherapy practices, with an average waiting time of 19.9 weeks for treatment initiation [21, 22]. Consequently, patients may not seek therapy at all due to long waiting periods [23, 24]. Access to alternative psychological support, such as primary and secondary prevention, is limited in the German healthcare system [25]. Considering the high prevalence of mental illness and the pandemic’s impact, it is crucial to provide more accessible psychological help, particularly through low-threshold interventions that compensate the long waits and accessibility challenges. Hence, our research team developed a low-threshold program to enhance mental health in a broad population. By combining clinical and positive psychological approaches, a new form of low-threshold intervention might be created.

For a long time, psychology focused on curing existing psychopathologies. Positive psychology has shifted the focus from dealing only with the negative aspects of life to further improving positive qualities [26]. In this context, the consideration of positive emotions plays a relevant role. The broaden-and-build theory points out that positive emotions can expand the repertoire of thought and action and build up lasting personal resources [27, 28]. Following these considerations, it seems to be useful to promote the experience of positive emotions to build up such resources. In the past decades, *positive psychology interventions* [29] based on the principles of positive psychology were developed to promote positive emotions, behavior, or cognitions [30]. Meta-analyses support the efficacy of positive psychology interventions for improving mental health and well-being [30–33].

However, most studies so far have examined individual exercises, but rarely entire holistic training programs. Mostly, such training programs were designed for specific target groups. The first training program of this kind was developed to increase happiness and life satisfaction in college students. It extended over six weeks and was able to significantly increase happiness of the participants [34]. Positive psychology training programs have also successfully been conducted with patients with various physical symptoms or diseases: Positive effects on pain perception and depressive symptoms as well as an increase of positive affect were found [35–37]. Furthermore, it was explored how positive psychology interventions can be used as a psychotherapy program: Positive psychotherapy was able to reduce mild to moderate depression [38]. A similar approach was chosen in a study by Chaves and colleagues in patients with major depression or dysthymia whose clinical symptoms and well-being were shown to improve [39].

Overall, the literature supports the effectiveness of positive psychology training programs. However, as described above, research so far has focused on testing the effectiveness of such trainings in specific target groups or individual exercises, but not as a holistic training program in the general population. To contribute to closing this research gap, we developed the Positive Emotions Training (PoET) which refers to the promotion of several positive psychology constructs and the improvement of mental health. The development of PoET was based on existing literature on positive psychology treatments as well as long-term discussions among the research group about how to promote respective constructs. For the training, an online format was chosen so that it could be conducted in larger groups despite the prevailing COVID-19 pandemic.

We decided to test PoET as an intervention and the methodical approach in a feasibility study. The main goal of this study was to evaluate the practicability of PoET and examine whether it might be an effective intervention to promote mental health. First, we hypothesized that participants receiving PoET would show a significant increase in positive mental health factors one month after the second training day (Hypothesis 1). Second, we expected to find a significant decrease in depression, anxiety, and stress symptoms one month after the second training day (Hypothesis 2). Third, we hypothesized participants in the control group not to show any significant changes in this regard (Hypothesis 3). In addition, we examined whether there might be a significant increase in optimism, gratitude, happiness, resilience, and self-efficacy for participants of PoET in comparison to the control group.

## Methods

### Recruitment

To advertise the PoET, a flyer was distributed on social media and two articles were published in newspapers. Those interested could contact an email address to receive further information about the training and to apply. Participants for the control group were also recruited via social media. As PoET is a low-threshold intervention, there were no exclusion criteria for participation and no screening for mental illness before the start. Any person older than 18 years could participate. Psychology students could get VPN hours as compensation. Overall, nearly 600 people were interested in participating. Because the number of psychologists to conduct training sessions was limited, not everyone got the possibility to participate. Places in the intervention study were not randomly allocated, but according to the principle “first-come, first-served”. Participants of the control group did not receive any treatment initially, but were offered to participate in one of the next PoETs.

### Participants

A total of *N* = 174 participants completed the registration process and baseline steps of the study and were finally offered to participate. Out of those, *n* = 100 were allocated to the PoET intervention group and *n* = 74 were allocated to the control group.

#### PoET

Since we were interested in the specific outcome effects of the training, only participants who took part at both training days and completed all data sets were considered in the analyses. *N* = 100 subjects took part in the first training session, of which *n* = 92 completed the first questionnaire. Up to the second session, there was a drop-out of *n* = 25 (27.17%). *N* = 1 (1.09%) did not fill out the last questionnaire. After another *n* = 11 (11.96%) data sets were removed because of missing values, *n* = 55 (59.78%) data sets were used for calculations. Participants in the PoET group were between 22 and 76 years old (*M* = 45.98, *SD* = 15.97) and *n* = 49 (89%) of the group were female. 12 participants were college students (22%), while *n* = 35 (64%) were employed and *n* = 8 (15%) were retired or classified themselves as homemakers. *N* = 39 participants were in stable relationships or married (71%).

#### Control

The final sample consisted of *n* = 46 persons because participants who did not complete the questionnaires at all three assessment points (*n* = 28, 37.84%) were excluded. Participants were between 19 and 68 years old (*M* = 35.13, *SD* = 15.37) and *n* = 35 (76%) of the group were female. *N* = 17 participants were college students (37%), *n* = 24 were employed (52%) and *n* = 5 (11%) were retired, in an apprenticeship or considered themselves homemakers. *N* = 28 participants were in stable partnerships or married (61%).

The two groups differed significantly in their age (*t*(99) = 3.459, *p* < .001).

### Development and description of PoET

For the development of PoET, a systematic literature analysis was first carried out within the context of the project “On the Role of Positive Emotions in the Promotion of Mental Health - An Experimental Study” to collect various positive psychology constructs and exercises for the promotion of positive emotion experience. Over a period of three years, these results were discussed in context of the Annual Congress for Psychotherapy in Bochum and a list of about 20 important positive psychological constructs was developed. A further literature analysis of the existing possibilities for promoting these constructs was conducted. Based on their potential regarding feasibility and effects on mental health, eleven constructs were selected by the team and included in the training. Exercises from already existing literature were compiled and own exercises were developed afterwards. After that, the exercises and formal aspects of the training were tested in a pilot phase in January 2022. In the context of this pilot phase, an exemplary training session was conducted with a group of volunteers and feedback was collected as well as integrated into the general PoET concept.

The PoET conducted in spring 2022 consisted of two training days of 3.5 h each, one week apart. The training was carried out online via Zoom in groups of about 30 to 35 people and was led by two psychologists who were also part of the research team. A total of eleven lessons on positive psychology constructs were conducted: happiness, hope, optimism, humor, self-efficacy, gratitude, flow, meaningfulness, forgiveness, spirituality, and resilience. At the beginning of each lesson, the participants received a short theoretical input on the respective positive psychology construct. After that, exercises were carried out either in the large group, in groups of three to six people in breakout rooms or alone. Afterwards, there was a short discussion on how the participants experienced each respective exercise. Finally, participants were given at-home-exercises. In addition, the participants received a booklet with descriptions of the exercises and space for notes. During the training days, a PowerPoint presentation was used as visual support. Between the two training days, there was a so-called 7-day challenge which should motivate the participants to complete the exercises at home. Table 1 lists the positive psychology constructs and the respective exercises.

**Table 1:** PoET lessons and respective exercises

Lesson	Exercise during training	Exercise at home
Happiness	**Happy cards**: Creating cards with personal positive messages (alone)	**Happy playlist**: Creating a playlist with songs evoking happiness **Happiness diary**: Daily answering of three questions (What made me happy today? What did I do well? How does it make me feel?)
Hope	**SMART goals**: Defining goals using the SMART function and discussing those goals in small groups	/
Optimism	**Best Possible Self exercise**: Thinking about one’s own best possible outcome in three areas (personal, relationships and work) and discussing those in small groups	/
Humor	**Funny group picture**: Taking a screenshot in breakout rooms while doing something funny	**Jokes/Cartoons**: Daily research for cartoons or jokes
Self-efficacy	**Self-reflection of positive characteristics in small groups**: Appearance, relationships, personality, external perception, successes, daily tasks	**Positive self-statement**: Formulating a short personal message and visualizing it in one’s daily life
Gratitude	**Exchange about today’s gratitude**: in small groups	**7 days of gratitude**: keeping a daily gratitude diary
Flow	**Exchange about the experience of flow in small groups**	/
Meaningfulness	**Stones in the water**: Thinking about one self’s positive influence on others and discussing it in small groups	**Pile of memories**: Creating cards about positive memories and reading those in calm/peaceful moments
Forgiveness	**Preparing a letter of forgiveness**: towards oneself or someone else (alone)	**Writing the letter of forgiveness**
Spirituality	**Loving Kindness Meditation**: in the large group	**Mantra Meditation**
Resilience	**Hands of competence**: Raising awareness of crisis skills via instructions in the large group. These skills are written in the fingers of a drawn hand outline.	/

### Measures

At all three assessment points, participants in the PoET and control group completed a battery of questionnaires. Socio-demographic data, questions about the experience of flow, current mood and stress, and exercise practice at home (in the PoET group) were assessed. In addition, the battery contained the German versions of ten validated questionnaires. The following seven questionnaires were relevant for this study.

#### DASS-21

The Depression Anxiety Stress Scale [40] was used to assess the negative emotional states of depression, anxiety, and stress. The questionnaire consists of 21 items. On a scale from 0 (“did not apply to me at all”) to 3 (“applied to me very much or most of the time”), participants could indicate their agreement with the items. The sum scores of the three subscales were used for calculations. Higher scores indicate worse mental health. A good internal consistency could be demonstrated for the three subscales depression (*α* = 0.89), anxiety (*α* = 0.75) and stress (*α* = 0.87).

#### PMH

The Positive Mental Health Scale [41] assesses emotional and psychological aspects of wellbeing across nine items. On a scale from 0 (“I do not agree “) to 3 (“I agree”), participants indicate their agreement with the items. Sum scores were formed for the calculations with higher scores indicating greater positive mental health. For the current sample an internal consistency of *α* = 0.91 was found.

#### LOT-r

The Life Orientation Test-revised [42] assesses the dispositional optimism. This questionnaire consists of 10 items. Of them, six capture dispositional optimism, while four items are filler items. On a scale from 0 (“strongly disagree”) to 4 (“strongly agree”), agreement with the items could be indicated. Higher values represent a higher level of dispositional optimism. The sum score was used for the calculation after three negatively formulated items have been inverted. In this sample, we found an internal consistency of *α* = 0.77.

#### GQ-5

The Gratitude Questionnaire [43] measures gratitude across five items. On a scale from 1 (“strongly disagree”) to 7 (“strongly agree”) agreement with the statements can be indicated. A scale mean was used for the calculation with higher scores indicating a more pronounced sense of gratitude. A Cronbach’s alpha of *α* = 0.82 could be found in the current sample.

#### SWLS

The Satisfaction with Life Scale [44] assesses global subjective happiness across five items. Participants were asked to rate their agreement with the items on a scale from 1 (“strongly disagree”) to 7 (“strongly agree”). The sum score was used for analysis with higher values indicating a happier person. For the current sample an internal consistency of *α* = 0.90 could be proven.

#### NGSE

The New General Self-Efficacy Scale [45] is an 8-item scale used to assess how much the participants believe in their ability to perform across a variety of situations. On a scale from 1 (“strongly disagree”) to 5 (“strongly agree”), agreement with the items can be indicated. Mean values were used for the calculation with higher scores representing higher self-efficacy. A Cronbach’s alpha of *α* = 0.89 could be found in this sample.

#### BRS

The Brief Resilience Scale [46] measures the participants’ ability to recover from stress despite significant adversity across six items. On a scale from 1 (“strongly disagree”) to 5 (“strongly agree”) participants could indicate their agreement with the items. The scale mean was used for the calculations with higher scores representing a higher resilience. For the current sample an internal consistency of *α* = 0.85 could be demonstrated.

### Procedure

#### PoET

**Fig. 1:**
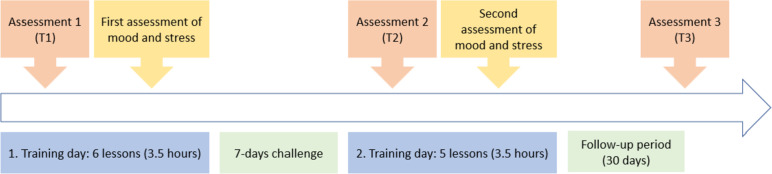
Chronological order of PoET procedure

Figure 1 shows the chronological order of PoET. To register for the training, all participants of the PoET group had to give their written consent. One day before the training, participants received the link to join the online meeting as well as the exercise booklet. The first training day started with all participants filling in the first questionnaire (T1). Afterwards, the participants were officially welcomed, and everyone had the opportunity to introduce themselves briefly. The rules for the training were explained and there was a short introduction to the topic of emotions which was followed by the lessons on happiness, hope, optimism, humor, self-efficacy, and gratitude. Thereafter, the 7-day challenge was explained, and the participants got a short preview on the contents of the second training day. Finally, participants’ mood and stress were assessed via two short items (on a 5-point Likert scale). The second day of training began with the completion of the second questionnaire (T2). Afterwards, participants were asked about their experiences during the 7-day challenge and the lessons on flow, meaningfulness, spirituality, forgiveness, and resilience were conducted. Then participants assessed their mood and stress in a short questionnaire. Finally, participants were asked which exercises they would integrate into their everyday life. Thirty days later, participants received an email with a link to the last questionnaire (T3).

#### Control

Participants of the control group only filled out the questionnaires at three assessment points. This happened in the same intervals as in the PoET group. They received the link to the questionnaires via email.

This study was approved by the Local Ethics Committee for Psychological Studies of Ruhr-University Bochum. All methods were performed in accordance with the relevant guidelines and regulations.

### Data analysis

The statistics program R Studio (version 2022.02.3) [47] was used for calculations. A two-factorial analysis of variance (ANOVA) with repeated measures was conducted to investigate changes in positive mental health factors (measured via PMH) in the PoET and the control group over the three assessment points. The two groups served as between-subject factor and the three assessment points as within-subject factor (2 × 3 mixed design). In the same procedure, possible differences in negative mental health, measured via DASS-21, were analyzed. The two-factorial analysis of variance with repeated measures was conducted for all three subscales depression, anxiety and stress. Bonferroni-adjusted pairwise comparisons between assessments and groups (time*group interactions) were performed. To test the prerequisites for the analyses, the Mauchly test for sphericity and the Shapiro-Wilk test for normal distribution were carried out. All prerequisites were met. The sample size was determined by power calculations which indicated that a sample of 61 would be sufficient for *p* < .05 with a power of 0.8 and an effect size of $\eta_p^2$ = 0.14.

In addition, we examined whether the individual constructs that were treated as lessons during the training had changed in the PoET group in comparison to the control group by conducting another two-factor analysis of variance with repeated measures. The two groups served as between-subject factor and the three assessment points as within-subject factor (2 × 3 mixed design).

The level of significance was set as *p* < .05 for all calculations. $\eta_p^2$ was calculated as effect size. According to Cohen [48], a $\eta_p^2$ of 0.01 can be considered small, 0.06 medium, and 0.14 a large effect. Missing data was not imputed. Data sets with missing data were excluded from the statistical analyses.

## Results

### Results at baseline

At T1, the two groups significantly differed from each other in their PMH scores (*p* = .032) with higher scores in the control group. For DASS-21 subscales, no significant differences were found between PoET and control group at baseline (all *p* values > 0.08). The complete baseline results can be found in Table 2.

**Table 2:** Baseline results for PMH and DASS-21 scores in PoET and control group

Measure	Group	Mean score	*SD*	Mean difference between groups	*p*
PMH	PoET	16.47	5.49	2.38	0.032*
	Control	18.85	5.44		
DASS-D	PoET	5.07	4.5	1.09	0.219
	Control	3.98	4.34		
DASS-A	PoET	3.58	3.18	1.1	0.086
	Control	2.48	3.2		
DASS-S	PoET	8.69	4.62	1.49	0.095
	Control	7.2	4.21		

*Note*. *PMH *Positive Mental Health Scale, *DASS-21 *Depression-Anxiety-Stress Scale, *DASS-D *Subscale “depression”, *DASS-A *Subscale “anxiety”, *DASS-S *Subscale “stress”

*p < .05, **p < .01, ***p < .001

### Effects of PoET on PMH scores

Descriptively, there was a higher increase in the PoET group PMH sum scores from T1 to T2 and T1 to T3 compared to the control group. While the PMH scores of the control group were significantly higher at T1, the PoET PMH scores approached the control at T2 and T3. The repeated measures ANOVA also showed a significant time*group effect (*F*(1.79, 177.34) = 3.385, *p* = .041; $\eta_p^2$ = 0.033) for T1. Despite the descriptive differences in the PoET group from T1 to T2 and T1 to T3, the Bonferroni-adjusted pairwise comparisons showed no significance for those differences (all adjusted *p* values > 0.193). There were no significant results for the control group either. All mean differences are presented in Table 3.

**Table 3:** Mean differences between assessment points (PMH)

Group	Time	Mean difference between assessment points	adj. *p*
PoET group	T1 – T2 T1 – T3 T2 – T3	1.93 1.78 -0.15	0.193 0.261 1
Control group	T1 – T2 T1 – T3 T2 – T3	0.31 0.37 0.06	1 1 1
*Note*. PMH = Positive Mental Health Scale *p < .05, **p < .01, ***p < .001.			

### Effects of PoET on DASS-21 scores

The repeated measures ANOVA showed significant time*group effects for the subscales DASS-D (*F*(1.87, 185.11) = 4.031, *p* = .011; $\eta_p^2$ = 0.046) and DASS-A (*F*(2, 198) = 5.233, *p* = .006; $\eta_p^2$ = 0.050). Bonferroni-adjusted pairwise comparisons showed that the PoET group DASS-D scores differed significantly from T1 to T2 (M_diff_ = -2.36, adj. *p* = .002, *d* = 0.62) and from T1 to T3 (M_diff_ = -2.56, adj. *p* < .001, *d* = 0.66). There were no significant differences between the assessment points in the control group (all adj. *p* > .507). It was the same for the DASS-A scores: The PoET group scores differed significantly from T1 to T2 (M_diff_ = -1.84, adj. *p* = .001, *d* = 0.65) and from T1 to T3 (M_diff_ = -2.05, adj. *p* < .001, *d* = 0.73). There were no significant differences between the assessment points in the control group (all adj. *p* > .624).

**Fig. 2:**
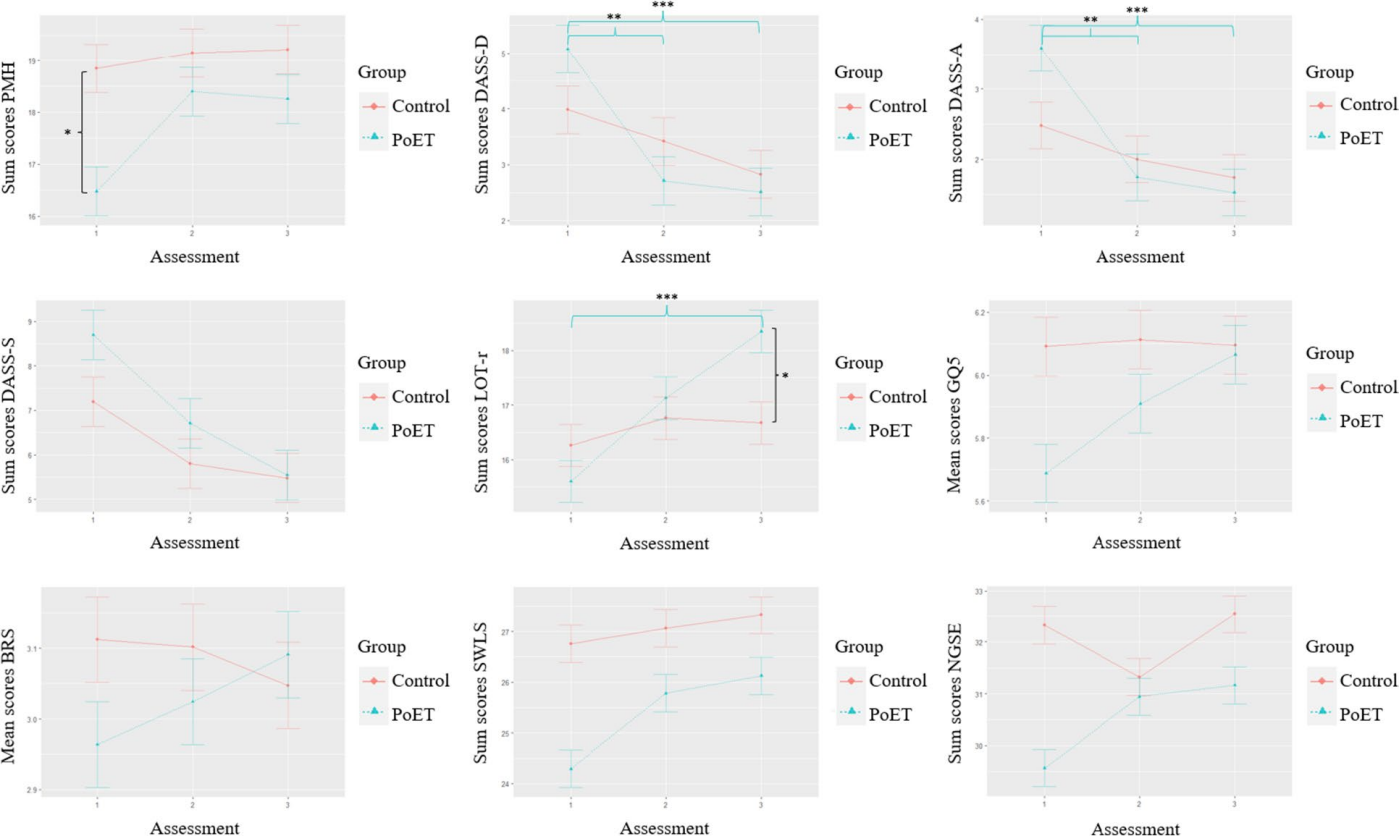
Changes in different measures for all assessment points and groups. Note. The blue lines depict significant changes in the PoET group. Significant differences between the groups at one specific assessment point are presented in black. PMH = Positive Mental Health Scale, DASS-21 = Depression-Anxiety-Stress Scale, DASS-D = Subscale “depression”, DASS-A = Subscale “anxiety”, DASS-S = Subscale “stress”, LOT-r = Life Orientation Test revised, GQ-5 = Gratitude Questionnaire, BRS = Brief Resilience Scale, SWLS = Satisfaction with Life Scale, NGSE = New General Self-Efficacy Scale. *p < .05, **p < .01, ***p < .001

 For the DASS-S there was a significant *time* effect (*F*(1.84, 182.48) = 19.504, *p* < .001; $\eta_p^2$ = 0.165). The DASS-S scores of the whole sample (independent from group affiliation) differed significantly from T1 to T2 (adj. *p* > .001) and from T1 to T3 (adj. *p* > .001). There were no significant time*group interactions. A graphic presentation of those findings can be found in Fig. 2.

### Effects of PoET on discussed positive psychology constructs

#### Optimism

The two-factor analysis showed a significant time*group effect (*F*(2, 198) = 8.742, *p* < .001; $\eta_p^2$ = 0.081) for the PoET group from T1 to T3 (adj. *p* < .001). There was also a significant difference between both groups at T3 (adj. *p* = .021) with higher scores in the PoET group.

#### Gratitude

 With respect to the graphic presentation of descriptive statistics, there was a high increase of mean gratitude scores in the PoET group from T1 to T2 and T1 to T3. Results of the conducted repeated measures ANOVA showed a significant time*group effect (*F*(1.78, 176.23) = 3.913, *p* = .026; $\eta_p^2$ = 0.038) which did not withstand the Bonferroni correction in any direction (adj. *p* > .091).

#### Resilience

Descriptive measures showed an increase of mean resilience scores in the PoET group from T1 to T2 and from T2 to T3. In the control group there was a slight decrease of mean resilience scores from T2 to T3. The repeated measures ANOVA showed no significant effects in neither of the two groups nor three assessments (all *p* values > 0.088).

#### Happiness

While the happiness sum scores in the control group graphically were relatively stable, there was a visible increase in the PoET group from T1 to T2. The repeated measures ANOVA showed a significant time*group effect (*F*(1.8, 178.45) = 3.560, *p* = .035; $\eta_p^2$ = 0.035) which did not withstand the Bonferroni correction (adj. *p* > .343).

#### Self-efficacy

The repeated measures ANOVA showed a significant time*group effect (*F*(2, 198) = 10.754, *p* < .001; $\eta_p^2$ = 0.098). The PoET and control group differed significantly at baseline (adj. *p* = .003) with *M* = 29.56, *SD* = 4.98 for the PoET group and *M* = 32.33, *SD* = 4.24 for the control group. Despite descriptive differences between the three assessments, the Bonferroni-adjusted pairwise comparisons showed no significance for those differences (all adjusted *p* values > 0.305).

A graphic presentation of the descriptive data for all relevant measures can be found in Fig. 2. All mean and sum scores of the different measures in this last part of the results are presented in Table 4.

**Table 4:** Mean scores of LOT-r, GQ-5, BRS, SWLS and NGSE

Measures	Group	Mean scores (*SD*)		
		T1	T2	T3
LOT-r	PoET	15.60 (3.90)	17.13 (3.77)	18.35 (3.39)
	Control	16.26 (3.42)	16.76 (3.38)	16.67 (3.75)
GQ5	PoET	5.69 (0.98)	5.91 (0.88)	6.07 (0.86)
	Control	6.09 (0.69)	6.11 (0.73)	6.10 (0.65)
BRS	PoET	2.96 (0.32)	3.02 (0.32)	3.09 (0.30)
	Control	3.11 (0.36)	3.10 (0.32)	3.05 (0.32)
SWLS	PoET	24.29 (6.21)	25.78 (5.87)	26.13 (6.10)
	Control	26.76 (4.86)	27.07 (4.24)	27.33 (4.50)
NGSE	PoET	29.56 (4.98)	39.95 (4.99)	31.16 (5.32)
	Control	32.33 (4.24)	31.33 (4.36)	32.54 (4.58)

*Note*. *LOT-r *Life Orientation Test revised, *GQ-5 *Gratitude Questionnaire, *BRS *Brief Resilience Scale, *SWLS *Satisfaction with Life Scale, *NGSE *New General Self-Efficacy Scale

## Discussion

The main goal of this study was to evaluate the practicability of PoET and to examine whether PoET might be effective to promote mental health in the general population. We hypothesized an increase in positive mental health factors (Hypothesis 1) and a decrease in depression, anxiety, and stress symptoms (Hypothesis 2) in PoET participants one month after the second training day. For the control group, we hypothesized that there would be no changes in these scores (Hypothesis 3).

Regarding the effectiveness of PoET, our results were mixed but definitely promising. Due to the fact that the groups were not randomly allocated, these findings should be handled with care.

According to the first hypothesis, there was a descriptive increase in positive mental health factors assessed with the PMH in the PoET group, but the differences were not significant in the statistical analyses so that the hypothesis could not be confirmed with the present data. These results are in contrast to previous research: While we found no significant changes in PMH scores of the PoET group, other studies showed positive effects of positive psychology interventions on (psychological) well-being [30–33]. When looking at descriptive values of the PMH, after a significant difference between the groups at T1 (with higher scores in the control), the PoET group scores increased so far that there no longer were significant differences at T2 and T3. These findings indicate that the training was effective in improving the PoET group’s positive mental health, however, this can only be described as a trend due to the lack of significance.

In this regard, it is important to refer to possible reasons for the absence of significance: One of them could be the differences of the PoET and control group. Since the groups were not randomly assigned, they differed significantly in age (*p* < .001) and in PMH scores at T1 (*p* = .041). Previous research has been able to find correlations between age and mental health [1, 49, 50]. Therefore, it would be necessary to examine PoET and its effects on positive mental health factors in a randomized-controlled trial to avoid significant group differences and allow analyses without confounding variables. Another contributing factor might be that this study did not record which exercises the participants continued to do at home. Thus, we were not able to backtrace the effectiveness of specific exercises. It is possible that some of the participants did not exercise at all within the 7-day challenge and after the training and that the boosting effect on their mental health was therefore not achieved. To ensure the training’s full potential, it would be helpful to assess the frequency and duration of exercising at home. Nevertheless, the results regarding the increase of PMH scores in the PoET group show that PoET as a low-threshold intervention was able to settle the significant differences between the two groups.

The second hypothesis could be partially confirmed with significant decreases of depression and anxiety symptoms assessed with the DASS-21 from T1 to T3. According to Cohen [48], the found effect sizes represent a medium effect. For stress symptoms, no significant effect was found but there was a descriptive decrease pointing in the same direction. The findings regarding Hypothesis 2 fit partially into the picture of previous research: Several meta-analyses could show significant effects of positive psychology interventions on depressive symptoms [30–33]. Like in our study, some of those meta-analyses also found small to moderate effects on anxiety symptoms. In contrast to our results, they also found significant effects on stress symptoms [32, 33]. Since stress measurements in this study showed an overall decrease in the PoET group among all three assessments, a long-term follow-up (e.g., three months) would have revealed possible significant long-term changes.

Overall, these results indicate that PoET is effective to reduce symptoms of depression and anxiety in the general population. It is still to explore, whether an adjusted version of PoET might have similar effects in a clinical population. In the future, based on these promising results, PoET should be further examined and evaluated in a randomized controlled trial.

According to the third hypothesis, there were no significant changes in the control group in neither of these measures, which suggests that the improvements in the PoET group can be attributed to the training itself and not to other possible environmental factors.

In addition to the three hypotheses, we also examined possible increases in other relevant dimensions in the PoET group in comparison to the control. These were optimism, gratitude, resilience, happiness, and self-efficacy. Solid significant changes could only be found in optimism with a medium effect size [48]. Increases in optimism in the PoET group were so high that their scores significantly exceeded the control group at T3. One possible reason for this effect might be the usage of the highly evaluated best possible self-method that is proven to be effective in many studies [51–53] and meta-analyses [54, 55]. Therefore, our findings match those of previous literature. Optimism is known to correlate with well-being under adverse circumstances [56], positive physical health measures [57] and less psychopathology [58, 59]. Hence, our results for optimism can be seen as particularly valuable.

For the other constructs (gratitude, resilience, happiness, and self-efficacy), no significant effects were found, but all of them increased descriptively, indicating that there were slight improvements. Since other studies have shown that positive psychology interventions are able to enhance these constructs [60–63], it can be assumed that the lack of significance is based on methodical issues. One of them might be that not all of the used exercises were as effective as the ones in the studies mentioned above. For self-efficacy, for example, due to the short time dimension of PoET, the exercise addressed self-efficacy more on a surface level, similar to self-confidence. Maybe this kind of exercise was not profound enough to change NGSE-scores. For happiness, one factor could be the used questionnaire, as the SWLS explicitly assesses life satisfaction and not happiness [44]. An alternative questionnaire that could be used in future studies is the Positive and Negative Affect Schedule (PANAS, [64]) whose measurements are probably closer to happiness than those of the SWLS. Another more general contributing factor could lay in the problem of group allocation, which was neither randomized nor matched, therefore resulting in group differences and difficulties regarding their comparability. However, it can be noted that there were some descriptive changes in the positive psychology constructs warranting further examination in a randomized controlled trial.

Besides these promising results, the main goal of this study was to test PoET’s feasibility regarding the general concept and the training circumstances. Implementing PoET as a two-session online training was experienced to be feasible by the research team as well as participants. Beforehand, we had considered whether to conduct the training within one session, two sessions or more. Although past positive psychology intervention studies often used five or more training sessions [34, 36, 39], we decided to keep our training concept as short as possible to minimize dropout rates and to facilitate the participation. Overall, participants reported that two sessions (3.5 h each) were perceived as the optimal solution because they were able to keep up their concentration during the sessions and integrate them into their daily life more easily than attending more sessions. Despite primary worries regarding the online format, conducting the training via Zoom had more advantages than disadvantages: The participation was possible from everywhere in Germany, difficulties regarding the arrival time and costs could be avoided, and the training could be conducted in large groups of about 30 people despite the ongoing COVID-19 pandemic. However, some participants experienced slight complications while logging in or retrieving the online questionnaire via Zoom. Those complications did not affect the training in general but were time consuming. In the future, it would be helpful to send detailed instructions on how to use Zoom before the beginning of the online session. Although the group size of approximately 30–35 persons was relatively large, it was perceived as fitting for this kind of training by participants as well as the research team. There was enough time for everyone to ask questions or share their experiences in the large group as well as possibilities to have more intimate conversations in breakout rooms in smaller groups.

### Limitations and future research

When interpreting the results, the following limitations must be considered. There were differences between both groups, that affected their comparability: At T1, the control group scored descriptively lower on the subscales of the DASS-21 and significantly higher (*p* = .032) on the PMH than the PoET group. In addition, the two groups differed significantly in their mean age (*p* < .001). This is critical as previous research has been able to find correlations between age and mental health [1, 49, 50]. Regarding the sample, it is also critical that there was no randomization, but rather a separate search for participants for the two groups, and that a large proportion of participants was female (PoET: 89%, control: 76%). High dropout rates of 45% in the PoET group and 37.84% in the control should also be considered. This can largely be attributed to errors in filling out the questionnaires or not being present at both training days. Finally, a limitation regarding the process of the questionnaires should be addressed: As no questionnaires were filled out after the end of the second training day, no statement can be made on how the training affected the mental health of the participants directly after completion. Thus, it could only be concluded that the training led to improvements in mental health at the beginning of the second training day and one month after the second training day.

Since the interest in participating in our study was tremendously high (nearly 600 people contacted us), future studies should address the urgent need for low-threshold interventions. Due to its simple structure, our PoET has the potential to build a foundation for a large-scale implementation. Future research is needed to test the effects of PoET on mental health in a randomized controlled trial. This would lead to more comparable groups when assessing the impact of the training on various positive psychological variables. As this research showed that PoET could significantly reduce depression and anxiety scores on the DASS-21 in the general population, it would also be interesting to investigate whether implementing the training can also improve depressive or anxiety symptoms in people with a diagnosed mental illness. Another interesting approach would be a comparison between an online and offline form of PoET to assess possible differences in the effectiveness.

## Conclusion

The main goal of the present study was to test the feasibility of a low-threshold intervention promoting positive emotions. The results showed that PoET in its recent form could be successfully conducted in the general population. In addition, significant effects on depression, anxiety, and optimism were found. Overall, PoET appears to have the potential to contribute to psychotherapeutic interventions. After further and clinical validation, PoET as a low-threshold treatment could be used in the area of prevention as well as during the long waiting periods before the beginning of therapy.

## Data Availability

The datasets used and analyzed during the current study are available from the corresponding author on request.

## Abbreviations

BRS: Brief Resilience Scale
DASS-21: Depression Anxiety Stress Scale
GQ-5: Gratitude Questionnaire Five Items Form
LOT-r: Life Orientation Test (revised)
NGSE: New General Self-Efficacy Scale
PMH: Positive Mental Health Scale
PoET: Positive Emotions Training
SWLS: Satisfaction with Life Scale
T1 – T3: Assessment 1–3
